# Does a low-carbohydrate diet impede endurance sports performance? Yes

**DOI:** 10.1016/j.ajcnut.2026.101268

**Published:** 2026-05-01

**Authors:** Louise M Burke, Timothy D Noakes

**Affiliations:** 1 Mary MacKillop Institute for Health Research, Australian Catholic University, Melbourne, Australia; 2 University of Cape Town, Cape Town, South Africa

**Keywords:** carbohydrate availability, sports nutrition, keto-adaptation, high performance athlete, exercise fuel utilization

## Abstract

The complex determinants of endurance sports performance vary according to exercise demands, the specific event, and the individual athlete. A key issue is the availability and integration of the oxidative use of muscle fat and carbohydrate (CHO) stores to supply energy. Current competition nutrition guidelines promote “high CHO availability,” using personalized strategies pre-, during, or between events to match glycogen and blood glucose supplies to the specific event demands. Alternatively, adaptation to a ketogenic low CHO high-fat (LCHF) diet makes fat the principal fuel, but impairs performance in some scenarios. Indeed, at oxidative thresholds, important in high-performance sport, metabolic pathways of fat oxidation provide a lower energy yield, leading to lower power/speed. Moreover, the consumption of CHO during exercise in keto-adapted athletes often enhances endurance capacity or performance. Therefore, the LCHF diet alone is suboptimal for endurance performance, as is as a one-size-fits-all approach to sports nutrition.

## Main Argument (Burke)

Endurance sports are continuous events lasting >30 min [1]. However, this definition fails to encompass the range of endurance events and myriad characteristics between and within sports that determine performance outcomes, even from the single perspective of nutrition support [2]. Performance determinants change not only with exercise characteristics (duration, intensity, etc.) [3], but with each iteration of the same event according to environmental conditions, terrain, tactics, nutrition support opportunities, and other factors [4]. Meanwhile, each athlete has unique characteristics including sporting “eliteness”/training status [5], sex [6], age, medical history and genetics. Such complexity prevents a single dietary approach from fulfilling its claims as the “the future of elite endurance sports,” as has occurred with the ketogenic low carbohydrate (CHO), high-fat (LCHF) diet [7]. Discussions around this claim have largely occurred with an oversimplistic, binary view of sports nutrition, best demonstrated within social media (the “Twitterverse”), but unfortunately not confined to this environment (Table 1 [4,[8], [9], [10]]). Although a debate might appear to force an absolute viewpoint about the “best” diet, making the “yes” case here only requires evidence that, in some circumstances, the LCHF diet fails to optimize performance (i.e., another approach is superior). This can be achieved while upholding the values of contemporary sports nutrition (Table 1).

**TABLE 1:** Comparison of the binary view of sports nutrition popularized in social media and the actual science and practice of sports nutrition

	Sports nutrition according to the “Twittersphere” (but unfortunately, not limited to social media)	The actual world of sports nutrition
Philosophy of “truth”	There is only 1 truth. The sports nutrition world involves 2 separate armies, each of which believe they are the holders of the truth. In contrast, the opposition is biased, unenlightened, and corrupted by industry. Every sports nutritionist and athlete must choose 1 allegiance.	There are many truths: individual approaches are needed for each athlete and each event, whereas knowledge and practice continue to evolve and innovate [4,8]. Sports nutritionists are excited by any performance-enhancing strategy—as long as it is safe, permitted in sport and evidence based [4].
Nutrition strategies for the endurance athlete	There are only binary choices. The athlete must choose between eating a high CHO diet on all occasions, as (wrongly) attributed to “current sports nutrition guidelines” or a ketogenic LCHF diet on all occasions.	Contemporary sports nutrition guidelines promote a personalized and periodized approach to sports nutrition strategies, including manipulating CHO *availability,* which may differ between and within different training sessions and events [8]. Official guidelines ceased recommending “high CHO” diets as an absolute or chronic strategy more than 2 decades ago but continue to be misrepresented [11].
Limitations to performance in endurance sports	A single limiting factor or performance determinant operates across all endurance events. It can be addressed with a singular strategy to achieve optimal performance.	Optimal performance in any endurance event is a complex and multifactorial concept. The athlete should develop a bespoke approach for each event, based on a nuanced understanding of their specific needs/goals, the features of the specific iteration of the event, and continual tweaking in response to insights gained in practice/training and previous events [4,8].
Examination of the literature	Studies that are heterogenous in design, interventions and methodological characteristics can be meta-analyzed into a single outcome. The average result represents a universal outcome.	Each study provides insights into the effects of different dietary strategies on sports performance as well as the suitability of study methodology to detect a finding. A “dashboard” approach maintains each data point to contribute to a more nuanced understanding of performance nutrition and study methodologies [9].
The value of testimonials	Observations of the nutrition strategies of a successful athlete—whether on a single occasion or as a description of chronic patterns—provide proof of their performance-enhancing effects.	Athletes sometimes develop successful nutrition strategies through trial and error but can also be successful despite their nutrition choices. Observations of novel strategies by elite athletes, especially when they grow in popularity, provide an incentive to undertake systematic investigation of the new practices.
Scientific collaboration	Sports scientists (or their followers) can win arguments by being loud, disrespectful and looking for “gotcha moments” which embarrass or discredit scientists on the opposing army. This may accelerate to online academic bullying [10].	Sports scientists can work together to better understand and implement the complexity of nutritional support for optimal sports performance. The science and practice of sports nutrition provides opportunities to investigate or implement a range of different (and often opposing) strategies.

### Rationale for optimizing performance via fuel use

Success in elite endurance sports is attained by the athlete who achieves the highest power outputs for the duration of the event and/or critical moments. A key focus is the athlete’s highest sustainable power output (or speed) known as critical power (CP); this physiological steady-state threshold is a composite of the athlete’s highest sustainable oxidative metabolic rate and economy [35]. High economy (speed/power produced per liter of oxygen consumed in oxidative metabolism) is a determinant of endurance performance, alongside a high peak aerobic capacity [VO_2_ (volume of oxygen) peak], high fractional utilization of this capacity (linked to metabolic/lactate threshold) and resilience (maintenance of these characteristics over the event duration) [35]. Although many factors, including nutritional status broadly, contribute to these characteristics, the availability and integrated use of the muscle’s fat and CHO stores for the oxidative regeneration of adenosine triphosphate (ATP) have been at the forefront of sports nutrition for the past 70 y [3].

High CHO availability refers to the capacity of the body’s CHO stores (principally glycogen and blood glucose) to meet the fuel needs of a specific exercise session [11]. Because liver glycogen is reduced by fasting and muscle glycogen can be depleted by prolonged and/or higher-intensity events, strategies to increase CHO availability have been developed for scenarios of high CHO demand [11]. These include muscle glycogen restoration/supercompensation by consuming appropriate amounts of CHO in the day(s) pre-event, a pre-event CHO-rich meal (restoring liver glycogen stores after an overnight fast), and within-exercise CHO intake [11]. Enhanced endurance performance is associated with sustained high rates of muscle CHO oxidation, and central nervous system (CNS) support for motor recruitment, pacing, and perception of effort [12]. Typically, high CHO availability is achieved by a combination of strategies, noting they both contribute to body CHO reserves and influence their absolute and store-specific rates of use [11,13], The amount of CHO intake required to achieve high availability is situation- and athlete-specific [14,15]. Importantly, sports nutrition guidelines promote high CHO availability only for endurance competitions and key training sessions which focus on performance quality or competition practice [11]. CHO availability may be unimportant for other workouts, whereas some training benefits may occur when sessions are undertaken with low availability [11].

The ketogenic LCHF diet (<50 g/d CHO, 75%–80% energy from fat) proposes an alternative approach to exercise fueling by allowing the relatively unlimited body fat stores to provide the main exercise fuel [16]. Keto-adaptation by athletes can achieve substantial (∼200%) increases in maximal rates (∼1.5 g/min) of exercise fat oxidation [[17], [18], [19]]. Muscle retooling of fat transport, uptake, and oxidation occurs in as little as 5–6 d [7], although it may take 3–4 wk to achieve other metabolic adaptations and restore perceptions of effort [20]. Keto-adaptation has been promoted as superior to the CHO-centric preparation for endurance competition [21], or at the worst, equivalent or immaterial [22]. Whether these claims can be substantiated is at the heart of this heated debate.

### Addressing (faulty) arguments that fuel sources for endurance performance are unimportant

**FIGURE 1:**
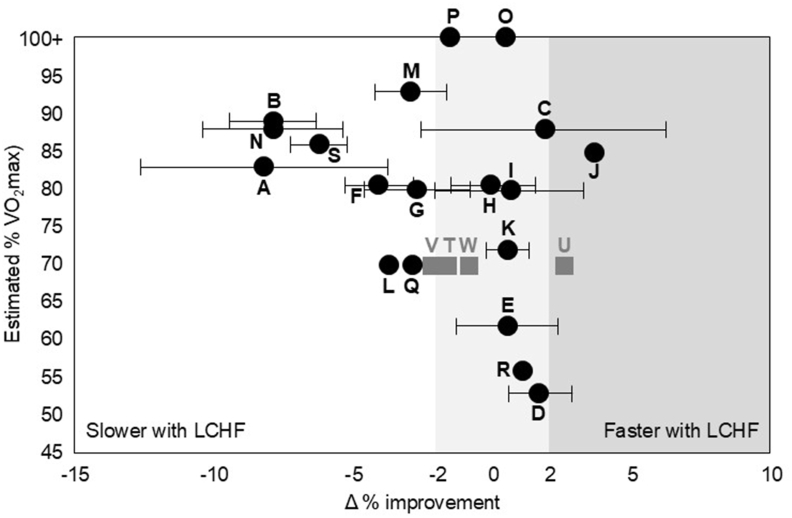
Updated dashboard [22] of changes in endurance performance after LCHF intervention in comparison to a condition with higher CHO availability. Data are expressed either as mean or mean ± SEM for time trial (time difference) or average power output (work rate) outcome. The threshold of worthwhile/substantial performance changes has been set at >2% to accommodate daily variability in exercise capacity/performance. Data points in this dashboard are taken from the following studies: A [18], B [17], C [24], D [25], E [26], F-1 [27], J [28], K [29], L [29], M [17], N [30], O–R [31], S [32], T–W [20]. CHO, carbohydrate; LCHF, low carbohydrate high fat; VO_2_max, volume of oxygen maximal.

The central argument opposing the current debate position is that systematic literature reviews/meta-analyses [22,23] have failed to find a difference in endurance performance between an LCHF dietary intervention and a “higher CHO availability” control. However, in elite sport, where statisticians do not determine the winner, accepting a null finding from the mean outcome of a heterogenous mixture of interventions, control conditions, study methodologies, and proxies for sports “performance” is as blunt as concluding that having one foot in the oven and the other in the freezer equates to a thermoneutral experience. As an alternative, we have constructed a visual dashboard of the available literature (Figure 1 [18,17,20,22,[24], [25], [26], [27], [28], [29], [30], [31], [32]]) that highlights study nuances rather than losing important details within a traditional meta-analytical approach [22]. A recent study [18] has been added to the original version (Figure 1: U, T, W, V). Readers are referred to the original dashboard [9] for full summarization of the heterogeneity of protocols—both in the nature and execution of the LCHF and “High CHO”/control comparisons. Our dashboard uses study heterogeneity to provide some insights into fuel issues for different types of endurance events:

1. Studies involving high-intensity aerobic exercise (85%–95% VO_2_max), similar to the CP/oxidative thresholds sustained in elite endurance competitions [17,18,[30], [31], [32]] are more likely to show inferior performance with LCHF conditions.
2. Studies that show small changes, likely within daily performance variability, typically involve lower exercise intensities (60%–75% VO_2_max) or supramaximal/anaerobically supported exercise [31].
3. Even at lower exercise intensities, effects of LCHF on endurance show substantial individual variability [25,26,28].
4. Studies showing equal/enhanced performance with LCHF condition are often confounded by methodological limitations including order effects [24,26], failure of the control condition to achieve high CHO availability [20,26,29], substantial noncompletion of LCHF trials [25], or substantial body mass loss/poorly matched dietary control [24,25].

With regard to the latter point, a full description of limitations or caveats in study design is noted in the original paper [9], including those in our own studies. We appreciate that many reflect the difficulty of undertaking such research, and the choice to compromise one aspect to maximize another. The critical confounding factor in this literature, however, is the measurement of *performance*. We recognize the challenge of replicating even a single endurance event, with sufficient reliability and ecological validity to detect worthwhile performance changes, within laboratory-based environments. Unfortunately, many studies actually investigate *exercise capacity*, using protocols that measure time to exhaustion, rather than *performance*. Although our dashboard uses a mathematical correlation to convert these to an equivalent “time trial” outcome [33], this action (and studies at large) fail to account for the complex physiological and psychological nature of pacing, or technical and tactical aspects of true sports competitions. Even when studies employ timed tasks, their execution may have poor precision and ecological validity in measuring performance. For example, running a 5-km “race” on a motorized treadmill while wearing apparatus attached to a metabolic cart involves distraction and a conscious action to change the treadmill speed in specific units [27]. Although there benefits to simultaneous collection of metabolic data, the downside is a reduction in performance sensitivity by preventing the athlete from achieving the brief or subtle pace changes that might determine the outcome of a real-world race. Reconstructing the intrinsic and extrinsic factors that underpin the motivation of authentic competition outside its environment is impossible.

### The case that LCHF can impair endurance performance

Three interrelated findings demonstrate that LCHF is suboptimal for *some* endurance sports. Our Supernova studies [17,18,30,32] have shown consistent evidence of compromised performance of real-world races involving higher-intensity (>80% $\dot{V}$O_2_max) exercise in elite race walkers who followed supervised LCHF diets (6 d–3.5 wk) compared with those who trained/competed with high CHO availability (Figure 1: A, B, N and S). This occurred despite equal training-induced increases in VO_2_max and the highest published rates of exercise fat oxidation (∼2 g/min).

We completely agree that fat oxidation can support high-intensity exercise. However, the stoichiometry of well-described biochemical pathways, empirically demonstrated 100 y ago [34], and in LCHF studies from our group [17,18,30,32], and others [28], states that ATP production from fat oxidation is ∼5%–7% less per liter of oxygen than CHO oxidation [35]. Therefore, this fuel choice is associated with a *loss* of exercise economy, similar in magnitude to the *improvements* in economy achieved by the carbon-plated foam-soled “super shoes” worn by distance runners during the recent breaking of every world record from 5 km to the marathon [36]. When exercise has already reached oxidative ceilings, lost ATP yield concedes to lower power/speed as a definite and fundamental flaw of fat oxidation. Interestingly, a subanalysis of our studies [37] showed an apparently larger performance decrement in the highest caliber walkers (medalists at major international events). This “throttling” of high-intensity submaximal exercise has been reported in other LCHF studies [28], including the original investigation in athletes [26]. Attempts to provide an alternative/additional fuel source for such bottlenecks, by acutely restoring muscle/liver glycogen [30], or consuming ketone ester supplements [32], are unsuccessful in fully restoring performance. Indeed, “fat adaptation” hits another metabolic roadblock, because it also involves muscle retooling to downregulate CHO metabolism [30,38,39] despite CHO availability.

The most recent study [20] contributes to the argument by showing that exercise capacity at 65%–70% VO_2_ peak was increased in both LCHF and control conditions by exercise CHO intake of 10 g/h CHO to prevent hypoglycemia. Why this function has been made the sole or pivotal reason for consuming CHO during exercise is unclear, because the wider literature demonstrates a variety of other roles including direct provision of muscle fuel, liver glycogen sparing, and CNS support [12], which differ in importance according to the specific scenario. Protocols to achieve high CHO availability for a specific scenario are also unique but are usually more effective when strategically combined [40]. Although the study concluded that “high CHO” exercise capacity was similar to LCHF [20], true high CHO availability involving a pre-event meal and higher exercise intakes of CHO to provide muscle substrate was not tested. Crucially, for the terms of this debate, consuming even small amounts of CHO improved cycling endurance compared with LCHF alone [20]. Because the principle of LCHF is extreme CHO restriction to remove CHO dependence [16], surely this is proof that LCHF in its singularity impairs endurance performance? A literal case of “having your cake and eating it too?”

Final evidence comes from a case study involving an elite Ironman triathlete who consumed larger (60 g/h) CHO doses during quality workouts within a training block, before performing various performance trials involving different exercise domains with similar CHO support [31]. Performance was improved compared with previous trials undertaken with LCHF alone (although, again, not directly compared against full strategies of high CHO availability), under conditions in which CHO likely played a metabolic role as well as providing CNS support [31]. Specifically, and predictably, 20-km cycling (sustained high-intensity exercise) was enhanced, with minimal impact on a 100 km (moderate intensity) or supramaximal tests [31]. Again, LCHF was shown to be suboptimal unless modified according to the rules it decries.

In conclusion, the literature shows, with sound theoretical underpinning, which LCHF alone is not optimal for at least some types of endurance sports. Therefore, the stated argument of this debate can be clearly defended. However, the science and practice of sports nutrition deserve better than the binary approach summarized in Table 1. Endurance athletes will benefit from a more inclusive and collaborative approach in which sports scientists systematically and strategically tackle iterations of protocols that manipulate higher CHO availability and/or high capacity for fat oxidation to find bespoke solutions.

## Refutation (Noakes)

Our goal for the past 4 y has been to establish whether muscle glycogen and high rates of CHO oxidation are obligatory for human performance especially during prolonged exercise. On the basis of, the principle that science advances by disproof of hypotheses [41], we completed a series of randomized controlled trials (RCTs) specifically designed to disprove our bias. Our findings establish that muscle glycogen is not obligatory for human exercise performance. Whereas, during prolonged exercise, blood glucose oxidation becomes an increasingly important obligatory CHO fuel.

If muscle glycogen is not an obligatory fuel for exercising humans, then the macronutrient composition of the pre-exercise diet is unimportant. I concluded “the macronutrient content of the habitual pre-exercise diet consumed for >4–6 wk has no influence on subsequent exercise performance.” Despite this evidence, Professor Burke continues to assert that: “Enhanced endurance performance is associated with sustained high rates of muscle CHO oxidation and central nervous system (CNS) support for motor recruitment (etc.).” Our studies establish that only the second part of that statement is partially correct. Interestingly she now acknowledges that: “We now completely agree that fat oxidation can support high-intensity exercise.” Her 2 beliefs appear contradictory.

Professor Burke argument for the superiority of the high-carbohydrate low-fat (HCLF) diet rests on the following: first, she suggests that there are 2 distinct groups of scientists, one of which “upholds the values of contemporary sports nutrition (Table 1)” whereas the other makes “claims that have largely occurred with an oversimplistic, binary view of sports nutrition best demonstrated within social media (the Twitterverse”), but unfortunately not confined to this environment (Table 1).” We publish our work in peer-reviewed journals and have produced the a comprehensive, evidence-based textbook arguing for the widespread medical value of an LCHF diet [42]. At the same time, her Table 1 excludes any reference to the persuasive role that industry plays in promoting a special role for CHO supplementation on exercise performance [43].

Second, she claims we are guilty of promoting a “single dietary approach” as the “future of elite endurance sport.” But this ignores her own decades-long efforts promoting CHO as the single dietary option for elite athletes [43]. Statements like: “‘Fat adaptation’ for athletic performance: the nail in the coffin?” [44] and “CHO fuels are the predominant and critical substrate for the working muscles, and the availability of CHO … rather than fat wins gold medals” [2, p.2] might suggest a distinctly “oversimplistic, binary view of sports nutrition.” Table 3 in [45, p.1] published in 2019 advises exclusively high CHO diets (CHO-loading) and CHO ingestion for all athletes competing in any running event from 21.1 km to the ultramarathon (>42 km). Her sole reference to the LCHF diet is dismissive: “such adaptations (to the LCHF diet) have been shown to impair exercise performance …. probably limiting the utility of the high-fat, low CHO diets to selected individuals, events or scenarios” (p.8).

Third, her inference that my narrative review [22] promotes “keto-adaptation as superior to the CHO-centric preparation for competition… or at the worst, equivalent or immaterial” misquotes me. I wrote: “Here, I provide the historical and modern evidence showing that the blood glucose concentration—reflecting the liver glycogen rather than the muscle glycogen content—is the homeostatically-regulated (protected) variable that drives the metabolic response to prolonged exercise. If this is so, nutritional interventions that enhance exercise performance, especially during prolonged exercise, will be those that assist the body in its efforts to maintain the blood glucose concentration within the normal range” (p.1). Our subsequent work has confirmed this interpretation as correct.

Fourth, in her limited response to our previous debate in 2024, Professor Burke [9] provided an original figure which she has since updated as a “visual dashboard of the available literature” (Figure 1). That figure compares the effects of the LCHF or the HCLF diets on exercise performance in the studies she chose to include. Figure 1’s visual impact is dominated by 5 of Professor Burke’s own Supernova studies (top left), which reported the largest performance benefits of the HCLF diet. Professor Burke is correctly skeptical of the ability of laboratory studies to recreate the conditions “that might determine the outcome of a real-world race.” Yet she is rather less judicious of her Supernova studies, perhaps because they are critical for her argument. But those studies are not RCTs [46].

The risk inherent in all non-RCTs is that they contain unimagined and therefore uncontrolled confounders. Thus: “Other study designs, including nonrandomized controlled trials, can detect associations between an intervention and an outcome. But they cannot rule out the possibility that the association was caused by a third factor linked to both intervention and outcome” [47, p.201]. In contrast our research includes only RCTs, all specifically designed to disprove our bias/hypothesis that ketogenic diets “are beneficial for exercise performance.”

**FIGURE 2:**
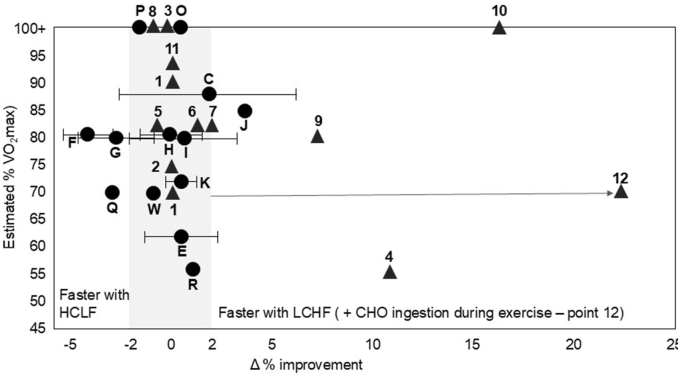
Modification of Figure 1 to include all published studies comparing the effects on exercise performance of LCHF vs. HCLF diets, excluding non-RCT studies like the Supernova series. Details of the additions and removals of identified studies can be found here at: https://thenoakesfoundation.org/research-rct-randomized-controlled-trial-lchf-low-carbohydrate-high-fat-diet-vo2max-maximum-oxygen-consumption/. CHO, carbohydrate; HCLF, high-carbohydrate low-fat; LCHF, low carbohydrate high fat; VO_2_max, volume of oxygen maximal; RCT, randomized controlled trial.

The Supernova studies also examined only one form of exercise lasting ∼42 min whereas our studies lasted from minutes to hours. To claim that her limited studies prove the superiority of the HCLF diet for all sporting events lasting from minutes to hours (ultramarathons) is not the best use of scientific evidence. I have revised this figure in panel B to exclude these non-RCTs and also include other published studies overlooked by Professor Burke. Clearly, the Supernova non-RCT studies are outliers. From this perspective, the claim that high CHO diets produce superior athletic performance during all forms of exercises at any intensity seems unsupported. In contrast, the regular ingestion of small amounts of CHO (10 g/h) during more prolonged exercise (point 12 in Figure 2) dwarfs any potential effects of the habitual high CHO diet that Professor Burke encourages.

## Rebuttal (Burke)

Unfortunately, sports nutrition continues to be misunderstood. Here, I state clearly that the “high CHO diet” was retired as a principle of sports nutrition guidelines decades ago [11]. Modern practitioners focus on CHO *availability,* periodizing and personalizing intake according to acute exercise goals, and integrating scenarios of deliberate increases or restrictions in CHO intake, plus situations of optional intakes. Indeed, my own recent clinical practice includes assisting a Vuelta cyclist to consume high rates of CHO on-bike, but keto-adapting an ultradistance swimmer for a 35-h ocean swim from Italy to Albania.

Perhaps I also misunderstood the original claim of the LCHF diet: removal of the need for CHO support for exercise. Yet, both our original LCHF dashboard and the Noakes’ update demonstrate *enhanced* endurance performance with the addition of CHO to LCHF. Obviously, LCHF itself is suboptimal. Stabilizing glycemia represents 1 benefit but neglects numerous others, including choosing the most economical (oxygen cost:energy produced) fuel for the muscle to deliver its maximal power/speed. The laws of stoichiometry, rather than my shortcomings, dictate greater energy yield from oxygen supply with CHO compared with fat oxidation; we’ve known this for over a century [7]. Thus, bespoke approaches to high CHO availability provide the greatest power production at maximal sustainable oxygen utilization during exercise. Others have confirmed better exercise economy with high CHO availability compared with LCHF [28], and recently, a dose–response with greater CHO intakes [48]. Therefore, can keto-adapted athletes salvage the inferior energy yield of fat oxidation by acutely manipulating endogenous and exogenous CHO sources pre/during their event? Alas, no. The price of fat adaptation is downregulation of the machinery underpinning maximal CHO oxidation: reductions in enzymes supporting glycogenolysis and tricarboxylic acid pyruvate entry [39], reduced muscle glucose transporter type 4 content [38], and missed opportunities to “gut train” for tolerance/absorption of exercise CHO intake [49].

Complex research often involves compromises, with both benefits and disadvantages. The Supernova racewalkers codesigned the studies which systematically searched for potential benefits of LCHF diets for their events. Although nonrandomized allocation of treatment provides an excuse to ignore our strikingly consistent results, it also removes unethical coercion of world-class athletes to follow an unwanted dietary treatment during periods of qualification or preparation for international competition. Importantly, our design standardized the “belief effect” by ensuring all athletes raced with self-chosen dietary strategies; this is a critical issue in maximizing performance efforts, mostly neglected in research. We chose 10,000 m distances, primarily to allow full recovery between races staged 3–4 wk apart, but also because they correlate highly with performance in longer athletic events [50], where they also represent speed at critical moments [51]. Previously, I have chosen not to draw attention to individual work, instead describing global concerns in study design/implementation in sports science research. However, if certain studies become the flagbearer for LCHF understanding, they deserve scrutiny. Here, I note that measurements of substrate oxidation via indirect calorimetry, under nonsteady-state conditions or with RER >1.0 [27,52], contravene basic scientific principles. Furthermore, mixing performance measurements with exercise capacity (Figure 2) fails to understand the clearly different magnitudes of change and their translation to sporting competition. Context matters!

## Disclaimer

This article series is designed as an Oxford-style debate. As such, participants are required to argue pro- and con- positions, even when that opinion may differ from their own. The views expressed in this debate do not necessarily reflect the opinion of the participants, *The American Journal of Clinical Nutrition*, or the American Society for Nutrition.

## Funding

Grants from the Australian Institute of Sport and Australian Catholic University funded all the studies conducted on low carbohydrate high-fat diets in sport.

## Declaration of generative AI and AI-assisted technologies in the writing process

No AI tools were used in the preparation of this paper.

## Conflict of interest

LMB has been an unpaid and short-term member of several Scientific Advisory Boards for Sports Nutrition Companies during selected periods of her career in sports nutrition: Gatorade Sports Science Institute Expert panel and Science in Sports. LMB has received travel grants from organizations related to both the carbohydrate and low carbohydrate high-fat (LCHF) industries to present on this topic. LMB has worked as a sports dietitian in elite sport for 45 years, supporting high-performance athletes to implement bespoke nutrition plans for their specific sporting pursuits. This includes assisting athletes to adopt periodized approaches to carbohydrate availability as well as specialized use of the LCHF diet for ultraendurance events. TDN is the author of low-carbohydrate nutrition books and a shareholder in The Nutrition Network.
